# *In Vitro* Inhibition of *α*-Amylase and *α*-Glucosidase by Extracts from* Psiadia punctulata *and* Meriandra bengalensis*

**DOI:** 10.1155/2018/2164345

**Published:** 2018-07-16

**Authors:** Yosief Kidane, Temesgen Bokrezion, Jimmy Mebrahtu, Mikias Mehari, Yacob Berhane Gebreab, Nahom Fessehaye, Oliver Okoth Achila

**Affiliations:** Department of Clinical Laboratory Sciences, Asmara College of Health Sciences (ACHS), Eritrea

## Abstract

**Background:**

This research assessed the* in vitro *antidiabetic activity and phytochemical constituents of the traditionally used medicinal plants,* Psiadia punctulata and Meriandra bengalensis*.

**Method:**

The leaves of both plants were subjected to cold extraction method using 70% ethanol and hot Soxhlet extraction using n-hexane, chloroform, methanol, and distilled water. The extracts were studied for their effect on glucose transport across yeast cells and inhibition of *α*-amylase and *α*-glucosidase enzyme activities. Thin-layer chromatographic analysis of ethanol extract was also undertaken.

**Results:**

The results of yeast glucose uptake assay revealed that extracts from both plants had a maximum increase in glucose uptake at the 25mM glucose concentration with a maximum dose of 2000*μ*g/ml plant extract. The ethanol extract of* P. punctulata* and aqueous extract of* M. bengalensis* showed a high activity of 68% and 96%, respectively, at 25mM and 2000*μ*g/ml of glucose and extract concentration.* P. punctulata *exerted peak inhibition activity of *α*-amylase of 37.5 ± 3% mg/dl (IC_50_ = 0.523 mg/dl) for methanol and distilled water extract at 0.5 mg/dl, respectively.* M. bengalensis* methanol extract exhibited the highest inhibition activity of 38 ± 8 % mg/dl (IC50 = 0.543 mg/dl) at 0.5 mg/dl. In the *α*-glucosidase inhibition assay, the methanolic extract of* P. punctulata *exhibited the highest inhibitory activity of 17.29 ± 9% mg/dl (IC_50_ = 0.761 mg/dl) at 0.5mg/dl. The chloroform extract of* M. bengalensis* had the highest inhibitory activity of 30 ± 5% mg/dl (IC_50_ = 0.6mg/dl) at 0.5 mg/dL. Phytochemical analysis of the different extracts of* P. punctulata* and* M. bengalensis* revealed the presence of flavonoids, alkaloids, tannins, saponins, phytosterols, and carbohydrates. Thin-layer chromatography analysis of ethanolic extract of both plants indicated presence of 15 and 17 spots for* P. punctulata* and* M. bengalensis* respectively.

**Conclusion:**

* P. punctulata* and* M. bengalensis* extracts have moderate inhibitory activity against pancreatic *α*-amylase and relatively low inhibitory activities against *α*-glucosidase. The observed effects may be associated with the presence of flavonoids, saponins, and alkaloids. Additional* in vivo* analysis, toxicological studies, isolation, and structural characterization of the phytomolecules identified in this study and molecular docking studies should be undertaken.

## 1. Introduction

Diabetes Mellitus (DM) is a complex metabolic disorder characterized by abnormal secretion and/or activity of insulin. Typical abnormalities of the disease include alterations of carbohydrate, protein, and lipid metabolism which manifest in a variety of complications including chronic hyperglycaemia, weight loss, polydipsia, polyuria, lethargy, and several macrovascular and microvascular complications [1]

At present, Diabetes Mellitus figures among the top ten killers worldwide. According to the International Diabetes Federation (IDF), the disease affected 285 million people in 2010, a figure projected to increase to 439 million by 2030 [2, 3]. The increasing prevalence presents a challenge because most of the pharmacotherapeutics/medicines indicated for the disease are not cures. In addition, allopathic drugs are costly, have limited tolerability, and have a range of adverse effects such as hypoglycemia and weight gain with sulfonylureas, potential liver toxicity with thiazolidinediones, and skin rash with insulin injection [4].

These shortcomings have encouraged a search for alternatives with herbal formulations gaining prominence, a fact underscored by a 1980 recommendation by the World Health Organization (WHO) on the need for scientific research on traditional herbal medicines [5]. To date, ethnobotanical studies of traditional herbal remedies with antidiabetic activity have identified more than 1200 plant species [6]. Documented activities of phytocompounds include stimulation of insulin release from pancreatic ß-cells, inhibition of the activity of glucose absorption in the gut, modulation of glucose, and reduction of end-stage glycation products, among others [7–9].

The fact that ethnopharmacology can be leveraged to direct and optimize the search for plant derived antidiabetics is evidenced further by the fact that Metformin, a first-line antidiabetic drug, was developed from a biguanide synthesized from* Galega officinalis* (French lilac) [10]. In addition, multiple studies have demonstrated that specific phytocompounds have comparable activity to *α*-amylase and *α*-glucosidase inhibitors such as Acarbose, Voglibose, and Miglitol [8–10].

In this study, we assessed the in vitro antidiabetic activity, specifically inhibition of *α*-amylase and *α*-glucosidase, of the plants* Psiadia punctulata (DC) Vatke* (family: Asteraceae) (locally named as Tsehay ferhet) and* Meriandra bengalensis (Koenig ex Roxb.) Benth* (family: Lamiaceae) (locally named as Mezaguf /nhba).* Psiadia punctulata *is mostly found in some east African countries including Eritrea, Saudi Arabia, and North East India. The plant is used in Eritrea primarily to treat ‘Gonfi' (febrile disease) but recently its antidiabetic effect has been reported in some literature [11]. In Eritrean pharmacopeia,* Meriandra bengalensis (M. bengalensis)* is indicated for hypertension, infection, hepatitis, malaria, and diabetes [12]. The plant is mostly found in the Southern region around Degerra Valley, near Segenaiti in Eritrea. Qualitative analysis of phytocompounds was also undertaken.

## 2. Materials and Methods

### 2.1. Reagents and Chemicals

The following chemicals were used in the study: chloroform (Blulux laboratories Product N^o^: CO1120, Batch N^o^: 35563), n-hexane (BDH laboratory Supplies Lot I 957476036, Prod 284884U), methanol (VWR International Ltd., Lot K36105570 626, Prod 101585A), ethanol (El Nasr Pharmaceutical Chemicals Co., E0058111), distilled water (National Health Laboratory, Asmara, Eritrea), DMSO (BDH Lab Supplies, Prod 282164K, Lot K31165384-289), *α*-amylase, *α*-glucosidase, and 2-deoxy-d-glucose (2-DG) (Sigma Aldrich Chemical Co., USA). Unless stated otherwise, all the chemicals were obtained from Sigma Aldrich.

### 2.2. Collection and Preparation of Plant Extracts

The selected plants* P. punctulata *and* M. bengalensis* were collected from the outskirts of Asmara on the way to Flfl-Solomuna, 40 Km North of Asmara. All the samples were authenticated by a botanist from the Eritrean Institute of Technology (EIT). All plant samples were deposited in the Department of Clinical Laboratory Sciences, (CLS) Asmara College of Health Sciences (ACHS). The leaves of both plants were shade-dried and powdered mechanically with a pestle and mortar.

Hot extraction method was carried out using hot continuous Soxhlet apparatus involving increasing polarity of solvents namely n-hexane, chloroform, methanol, and distilled water. The cold extraction used 70% ethanol.

The solvents were removed under reduced pressure and controlled temperature by rotary evaporator (*Heidolph)*.The extracts were dried and stored in a clean glass bottle and kept at 4-6°C for further use of* in vitro *antidiabetic assays.

### 2.3. Antidiabetic Activity

The different plant extracts were tested for their effects on glucose uptake by yeast cells and their inhibitory effect against digestive enzymes (*α*-amylase and *α*-glucosidase).

### 2.4. Glucose Uptake in Yeast Cells

A 10% (v/v) suspension was prepared in distilled water after repeated washing of commercial baker's yeast. Into 1ml glucose solution different concentrations of plant extract were added and incubated together at 37 degrees Celsius. After 10 min of incubation to initiate the reaction, a yeast suspension was added; this was mixed by vortexing. The reaction mixture was further incubated for 60 minutes at 37 degrees Celsius. Then, the tubes were centrifuged and glucose was estimated in the supernatant spectrophotometrically. Metronidazole was taken as standard drug. The percentage increase in glucose uptake by yeast cells was calculated using the following formula:

(1) $$\begin{array}{c} \%\;\;Uptake=\frac{{Ab}_{c}\text{ - }Ab_{s}}{{Ab}_{c}}\times 100 \end{array}$$

where Abc_control_ is the absorbance of the control reaction (containing all reagents except the test sample) and Abs_sample_ is the absorbance of the test sample [13, 14].

### 2.5. Inhibition of *α*-Amylase Enzyme

This assay was carried out using a modified procedure of McCue and Shetty [15]. The plant extract was placed in a test tube and 0.02M sodium phosphate buffer (pH 6.9) containing *α*-amylase solution was added. This solution was preincubated at 25 degrees Celsius for 10min; later 1% starch solution in 0.02M sodium phosphate buffer (pH 6.9) was added and then further incubated at 25 degrees Celsius for 10min. The reaction was terminated by adding 2 ml, dinitrosalicylic acid (DNSA) reagent (40 mM, K^+^-Na^+^ tartrate 1 M, NaOH 0.4 M). The test tubes were then incubated in boiling water for 5 min and cooled to room temperature. The reaction mixture was diluted with distilled water and the absorbance was measured at 25 degrees Celsius at 540 nm using a spectrophotometer.

A control was prepared using the same procedure replacing the plant extract with distilled water. The *α*-amylase inhibitory activity was calculated as percentage inhibition:

(2) $$\begin{array}{c} \%\;\;Inhibition=\frac{{Ab}_{c}\text{ - }Ab_{s}}{{Ab}_{c}}\times 100 \end{array}$$

where Abc_control_ is the absorbance of the control and Abs_sample_ is the absorbance of the sample

### 2.6. Inhibition of *α*-Glucosidase Enzyme

The inhibitory activity was determined by incubating a solution of maltose substrate with Tris buffer pH 8.0 and various concentrations of plant extracts at 35 degrees Celsius. The reaction was initiated by adding *α*-glucosidase enzyme into the reaction mixture followed by incubation at 35 degrees Celsius. Then the reaction was determined by the addition of a colorimetric reagent (DNSA). A control was prepared using the same procedure replacing the plant extract with distilled water. The intensity of the color was measured at 540nm. Percentage inhibition (I %) was calculated by

(3) $$\begin{array}{c} \%\;\;Inhibition=\frac{{Ab}_{s}\text{ - }Ab_{c}}{{Ab}_{s}}\times 100 \end{array}$$

where Abc_control_ is the absorbance of the control and Abs_sample_ is the absorbance of the sample.

### 2.7. Phytochemical Analysis and Thin-Layer Chromatography (TLC)

Qualitative phytochemical analysis of n-hexane, chloroform, methanol, distilled water, and ethanolic extracts was undertaken according to the method described by Prashant Tiwari and colleagues [16]. Because ethanol showed a comparatively higher percentage yield, thin-layer chromatography (TLC) was used as a qualitative means of separating the various compounds present in the plant extract.

### 2.8. Preparation of the TLC Plates

TLC plates of thickness 0.25mm were placed in an oven at 100 degrees Celsius for 30 minutes to activate the silica gel. The plates were taken from the oven and kept at room temperature for 15 minutes. The dried filtrate, obtained from both plants, was dissolved in ethanol to a ratio of 1mg/ml using a calibrated microcapillary tube; a small drop of the ethanol extract of the plants was placed on the TLC plate, 3 cm (above the bottom). This spot was allowed to dry and the TLC plate was placed into the TLC chamber which was saturated with chloroform solvent carefully to have uniform solvent level. When the solvent reached 2 cm below the top, the plates were taken out of the chamber and detected with UV spectrophotometry. R_f_ values of the spots were calculated by:

(4) $$\begin{array}{c} R_{f}=\frac{\text{Distance}\;\;\text{travelled}\;\;\text{by}\;\;\text{the}\;\;\text{sample}}{\text{Distance}\;\;\text{travelled}\;\;\text{by}\;\;\text{the}\;\;\text{solvent}}. \end{array}$$

## 3. Data Analysis

All analysis was undertaken in triplicate and each experiment was repeated three times. Quantitative values were presented as means ± standard deviation (SD). One-way analysis of variance (ANOVA) was used to evaluate the statistical differences between different inhibitory concentrations followed by Tukey HSD* post hoc* test. Probit regression modelling was used to estimate the inhibitory concentration 50 (IC_50_). Statistical analysis was performed using statistical package for social sciences (SPSS), version 20.0 software, and Microsoft 2007) and SPSS version 16.0 for Windows 7. Differences at P < 0.05 were considered significant.

## 4. Result 

### 4.1. Percentage Yield

The percentage yields of crude extracts are given in Table 1. Both plants* M. bengalensis *and* P. punctulata *extracts showed highest yield in their cold extracts with a yield of 23.7% and 22%, respectively.

### 4.2. Glucose Uptake in Yeast Cells

The current research tried to assess the* in vitro* antidiabetic potential of two medicinal plants used in Eritrea for treating Diabetes mellitus.

**Figure 1:**
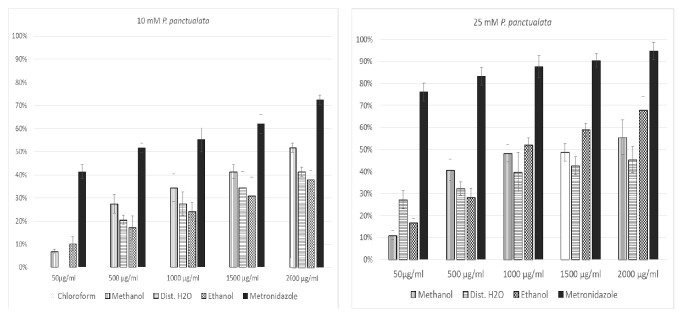
Glucose uptake in different concentration of plants extracts.

The selected plant extracts were studied for their effect on glucose uptake in yeast cells using different concentrations of glucose solutions. Yeast cells were incubated in glucose solution containing different concentrations of the two plants extracts and the glucose uptake was assessed. The different solvent extracts of both plants showed an increased activity of glucose uptake in yeast cells. As shown in Figure 1, the glucose uptake in yeast cells increased significantly with an increase in glucose concentration (50 *μ*g/ml, 500 *μ*g/ml, 1000 *μ*g/ml, 1500 *μ*g/ml, and 2000 *μ*g/ml) and plant extract concentration (10 mM and 25 mM).

The crude extracts for* P. punctulata*, however, showed almost no activity at 5 mM of glucose concentration. The n-hexane and chloroform extracts did not show any activity across all glucose concentrations measured.

**Figure 2:**
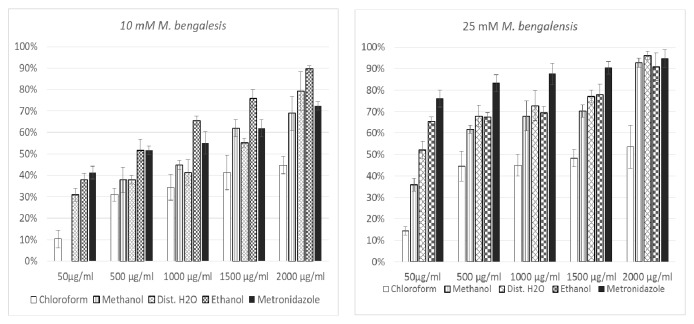
Glucose uptake in different concentration of plants extracts.

The plant* M. bengalensis* exhibited an increased glucose uptake activity for the five different extracts at the four different glucose concentrations measured (Figure 2).

At 25 mM of glucose concentration, all the four different plant extracts exhibited an increased activity ranging from 14.35 to 96.17% with the different doses of plant extracts ranging from (50 *μ*g/ml, 500 *μ*g/ml, 1000 *μ*g/ml, 1500 *μ*g/ml, and 2000 *μ*g/ml). The highest activity at 25mM of glucose concentration was recorded for the aqueous extract which was 96.17% at a maximum dose of 2000*μ*g/ml.

### 4.3. Inhibition of *α*-Amylase

**Figure 3:**
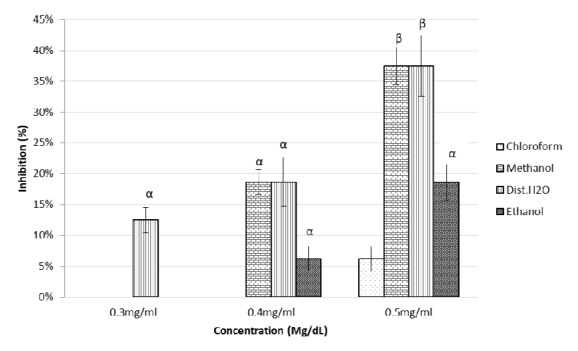
Inhibitory potency of* P. punctulata* extracts against *α*-amylase activity. The values are expressed as means ± SD., n=3. *α* and *β* compare the effect of different concentrations of a particular extraction solvent on *α*-amylase inhibitory activity and subsequent bars designated by different letters signify significant different inhibitory activity p < 0.05 (Tukey HSD test).

The* in vitroα*-amylase inhibitory activities of the five solvent extracts of* P. punctulata *and* M. bengalensis* were assayed. The results indicated that at the lowest concentrations of 0.1 mg/dl and 0.2mg/dl there was no inhibition activity (Figure 3).

The magnitude of inhibition depended on the nature of the extract and concentration used with group comparisons including negative control (NC) exhibiting a value < 0.05 for all extracts. The methanol and distilled water extracts of* P. punctulata *exerted peak inhibition activity of *α*-amylase of* 37.5 *± 3% and* 37.5 *± 5% for methanol and distilled water extract at 0.5 mg/dl, respectively. The concentrations at 0.4 mg/ml and 0.5 mg/ml differed significantly for both methanol (p < 0.023) and distilled water (p < 0.004) extracts. A lower inhibition rate of 16 ± 3% was observed for the same enzyme for ethanol extract. The IC_50_ values of methanol and distilled water extracts were 0.523 mg/dl and 0.543 mg/dl, respectively. (Table 2).

Inhibition of *α*-amylase by* M. bengalensis* extract increased with concentration and depended on the nature of the extract. Methanol extract exhibited the highest inhibition activity of 38 ± 4 % at 0.5 mg/dl. Ethanol and distilled water extracts demonstrated highest inhibition activities of 25 ± 2 % and 19 ± 4 %, respectively, at 0.5 mg/dl.

**Figure 4:**
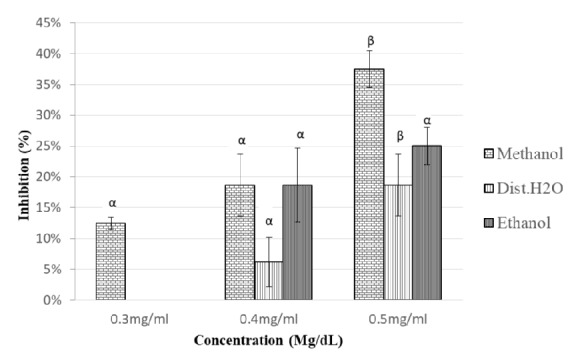
Inhibitory potency of* M. bengalensis* extracts against *α*-amylase activity. The values are expressed as means ± SD., n=3. *α* and *β* compare the effect of different concentrations of a particular extraction solvent on *α*-amylase inhibitory activity and subsequent bars designated by different letters signify significant different inhibitory activity p < 0.05 (Tukey HSD test).

The inhibition activities for the other concentrations of the extracts employed in the study are as shown in Figure 4. The inhibitory activity at 0.4 mg/dl versus 0.5 mg/dl for methanol and distilled water extracts differed significantly, p < 0.001 and p < 0.021, respectively, as shown in Figure 4. The IC_50_ for methanol, ethanol, and distilled water extracts was 0.543 mg/dl, 0.572 mg/dl, and 0.6 mg/dl, respectively (Table 2).

### 4.4. Inhibition of *α*-Glucosidase

**Figure 5:**
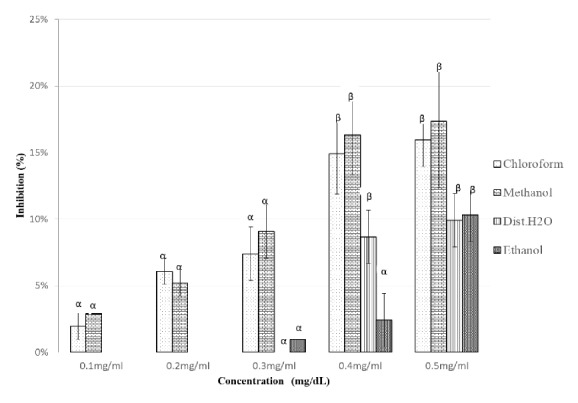
Inhibitory potency of* P. Punctulata *extracts against *α*-amylase activity. The values are expressed as means ± SD., n=3. *α* and *β* compare the effect of different concentrations of a particular extraction solvent on *α*-amylase inhibitory activity and subsequent bars designated by different letters signify significant different inhibitory activity p < 0.05 (Tukey HSD test).

In the current research, the different extracts of both plants were able to demonstrate some inhibitory activity against *α*-glucosidase when compared to NC. In the* P. punctulata *assay; the methanolic extract exhibited the highest inhibitory activity of 17.29 ± 9% at 0.5mg/dl. The chloroform extract had an inhibitory activity of 16%  ± 5%. The inhibitory activity differed significantly at concentration 0.3 mg/ml and 0.4 mg/ml for chloroform and methanol extracts, p < 0.014 and p < 0.021, respectively. The IC_50_ for methanol and chloroform extract was 0.761 mg/dl and 0.874 mg/dl, respectively. The other extracts had concentrations less than 10% (Figure 5).

**Figure 6:**
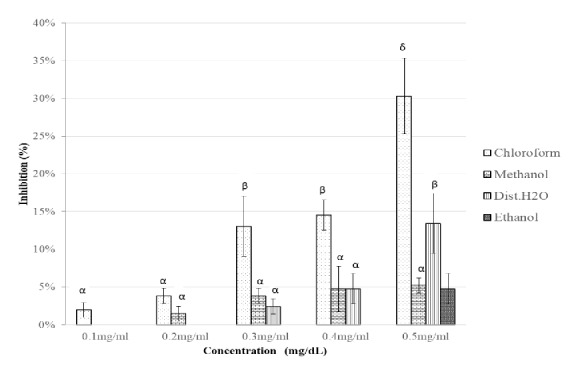
Inhibitory potency of* M. bengalensis* extracts against *α*-amylase activity. The values are expressed as means ± SD., n=3. *α*, *β*, and *δ* compare the effect of different concentrations of a particular extraction solvent on *α*-amylase inhibitory activity and subsequent bars designated by different letters signify significant different inhibitory activity p < 0.05 (Tukey HSD test).

In the* M. bengalensis* assay, inhibitory activity against *α*-glucosidase increased significantly with increasing concentration (0.1 – 0.5 mg/dL) for the chloroform extract with highest inhibitory activity of 30 ± 5% mg/dl observed at 0.5 mg/dL. A significant difference in the inhibitory activity of various extracts was observed at disparate concentrations of chloroform extract (0.4 mg/dl and 0.5 mg/dl, p < 0.001). An inhibitory activity of 13 ± 2% was observed for the distilled water extract (Figure 6). The IC_50_ for the chloroform extract was 0.599 mg/dl.

Both plants showed a weaker *α*-glucosidase inhibition compared to the results reported for inhibition of *α*-amylase enzyme activity.

### 4.5. Phytochemical Analysis

Preliminary phytochemical screening on the five different solvent extracts of* P. punctulata *and* M. bengalensis *demonstrated presence of different chemical entities. The aqueous extract of* P. punctulata* showed presence of maximum compounds like alkaloids, tannins, saponins, flavonoids, and carbohydrates. For the plant* M. bengalensis*, the highest number of chemical entities such as alkaloids, tannins, saponins, flavonoids, carbohydrates, and phytosterols were detected for the methanolic, aqueous, and ethanolic extracts (Table 3).

**Figure 7:**
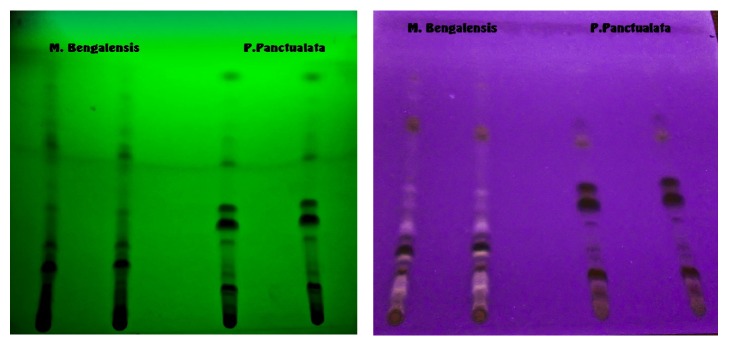
TLC reading at 254 and 336 nm of the plants* M. bengalensis* and* P. punctulata*.

Thin-layer chromatography (TLC) was performed on methanolic extract of both plants to assist in tracking the unknown phytoconstituents. The results indicated presence of 15 and 17 spots for* P. punctulata* and* M. bengalensis*, respectively, indicating the presence of a number of phytoconstituents (Figure 7).

## 5. Discussion

Multiple strategies have been devised or explored in the management of DM. Stimulation of Adenosine monophosphate-dependent protein kinase (AMPK) (Biguanides-Metformin); blockage of ATP-gated K^+^ channels in *β* cells (Sulfonylureas-Glipizide); stimulation of peroxisome proliferator–activated receptors activities (PPAR *ϒ*) (Thiazolidinediones-Rosiglitazone); and glucagon-like peptide-1 (GLP-1) (Exenatide-Byetta) modulation [17, 18]. The agents are directed at either enhancing insulin secretion, insulin sensitivity, or reducing glucose production by the liver.

Another important approach is directed at the management of post-prandial hyperglycemia (PPH) by inhibiting the activity of *α*-amylase (cleavage of 1, 4-*α*-D-glucosidic linkages in polysaccharides) and *α*-glucosidase (terminal-hydrolysis of 1, 4-*α*-D-glucosidic linkages in oligosaccharides). Reducing PPH is important given the fact that it can help in reducing advanced glycation end-products (AGEs) formation, a metabolite which has been identified as a major risk factor for cardiovascular complications in DM patients [19]. In particular, it has been suggested that pharmacotherapeutical inhibition of the activity of *α*-amylase and *α*-glucosidase may be beneficial to DM patients with impaired insulinotropic response, especially when used in combination with other oral hypoglycemic agents (OHA) [20]. Their potential in limiting weight gain or enhancing weight loss even in nondiabetic patients has also been proposed [21].

In the recent past, a lot of attention has been directed at elucidating the mechanism of action and phytochemistry of herbal extracts indicated for DM in traditional pharmacopeia [22]. Focus on the inhibitory activity of phytochemicals on *α*-amylase and *α*-glucosidase has particularly been popular. Interest in identifying pharmacologically active phytoconstituents which can inhibit *α*-amylase and *α*-glucosidase is premised on the claim that they have fewer side effects and are less expensive compared to synthetic pharmacotherapeutics like Acarbose and Miglitol [21, 22]. In this regard, and in keeping with this quest, we investigated two plants* in vitro*,* Psiadia punctulata* and* Meriandra bengalensis, *used in Eritrean pharmacopeia for DM management.

Methanolic and distilled water extracts of* P. punctulata *exhibited significant inhibitory activity against *α*-amylase at the highest concentration (0.5 mg/dl) of the extract used. The IC_50_ estimates were 0523 mg/dl and 0.543 mg/dl, respectively. The observed inhibitory strengths are comparable to those of similar studies [23–25]. The inhibitory activity of* P. punctulata* extracts on *α*-glucosidase was not as potent. The chloroform and methanol extracts had comparatively low inhibitory activity 16% and 17.29% and high IC_50_ values, 0.874 mg/dl and 0.761 mg/dl. It should be emphasized that* P. punctulata* is a member of* Asteraceae *family and some studies have shown that extracts from plants in this family can inhibit the activity of carbohydrate hydrolysing enzymes [25].

Qualitative phytochemical analysis of the analytes obtained from multiple extraction solvents detected several phytomolecules including flavonoids, saponins, tannins, alkaloids, and phytosterols. The TLC chromatogram of the methanolic extracts indicated presence of 15 and 17 spots demonstrating the presence of multiple phytomolecules. The presence of flavonoids, especially in chloroform, ethanol, methanol, and distilled water extracts, may account for the inhibitory activity observed. Flavonoids, heterogeneous group of plant polyphenols, have widely reported inhibitory activity against *α*-amylase and *α*-glucosidase in both* in vitro* and* in vivo* and* in silico *modelling studies [9, 26–28]. Importantly, some investigators have reported that there is a positive relationship between total flavonoid and polyphenol content and the ability to inhibit *α*-amylase and *α*-glucosidase [29]. Antiatherogenic effects of flavonoids have also been reported [30, 31]. The additional inhibitory activity observed for methanol and distilled water extracts of* P. punctulata* may be associated with the presence of other phytoconstituents like alkaloids and saponins. Saponins have been associated with suppression of fluid and glucose uptake at the brush borders [20]. Other studies have demonstrated that some plant extracts can enhance insulin secretion and insulin signaling in adipose and skeletal muscles [32, 33].

The inhibitory activity of extracts of* M. bengalensis* was also evaluated. According to the results obtained, methanol, distilled water, and ethanol extracts had significant inhibitory activity against *α*-amylase at 0.5 mg/dl. This fact is also evidenced by the IC_50_ estimates obtained 0.543 mg/dl, 0.572 mg/dl, and 0.6 mg/dl, respectively. Qualitative phytochemical analysis of the extracts obtained by the use of these solvents detected several pharmacologically active phytoconstituents, including flavonoids, tannins, and saponins. Therefore, the observed inhibitory effect may be attributed to these compounds (29-30). Further, only the chloroform extract exhibited significant inhibitory activity against *α*-glucosidase.

## 6. Conclusion

This is the first study to evaluate the inhibitory effect of* P. punctulata* and* M. bengalensis* against specific carbohydrate hydrolysing enzymes. According to the results obtained, the extracts from* P. punctulata* and* M. bengalensis* demonstrated moderate inhibitory activity against pancreatic *α*-amylase and relatively low inhibitory activities against intestinal *α*-glucosidase. Therefore, the antidiabetic effects of the two plants may be associated with the observed inhibitory activity against the specified carbohydrate hydrolysing enzymes. In particular, specific phytocompounds, including flavonoids, saponins, and alkaloids detected in the crude extracts, may be responsible for the observed activity. In this regard, the observed results may justify the traditional use of these plants in the management of post-prandial hyperglycemia in Type 2 Diabetes Mellitus (T2DM). However, it should be noted that results obtained* in vitro* are not necessarily confirmable by* in vivo* tests in appropriate animal models or in randomized clinical studies. In this regard, additional* in vivo* studies are warranted. The need to address toxicological issues is also pertinent. Further, elucidation of the mode of inhibition, isolation, and structural characterization of the phytomolecules and quantitative structure activity relationships (QSAR) using* in silico* modelling and other platforms for structural analysis should be undertaken.

## Figures and Tables

**Table 1:** Percentage yield in hot and cold extraction for different extracting solvents.

**Materials**	**Hot extraction**				**Cold extraction**
	*Extracting solvents*				
	*n-Hexane*	*Chloroform*	*Methanol*	*Dist.H*_*2*_ *O*	*Ethanol*
*Weight of dried and powdered plant leaves*	**30gm** %**w/w**	**30gm** %**w/w**	**30gm** %**w/w**	**30gm** %**w/w**	**50gm** %**w/w**

*P. punctulata*	10.4%	13.3%	15.9%	20.8%	22.00%

*M. bengalensis*	9.6%	12.5%	16.1%	21.2%	23.7%

**Table 2:** Estimated inhibitory concentration 50 (IC_50_) values for *α*-glucosidase and amylase inhibition for *Psiadia punctulata *and *Meriandra bengalensis*.

**Analyte **	**Inhibitory Concentration, IC50 (** **µ** **g/ml)**			
	*Psiadia punctulata*		*Meriandra bengalensis*	
	*α*-glucosidase	*α*-amylase	*α*-glucosidase	*α*-amylase
*Chloroform *	*0.874*	*-*	*0.6*	*-*

*Methanol*	*0.761*	*0.523*	*-*	*0.543*

*Distilled H*_*2*_ *O*	*-*	*0.543*	*-*	*0.572*

*Ethanol*	*-*	*-*	*-*	*0.599*

**Table 3:** Results of the qualitative analysis of the presence of specific phytoconstituents in difference extracting solvents.

**Phytomolecules**	**Method**	**Extracting solvents**				
		*n-hexane*	*Chloroform*	*Methanol*	*Dist.H*_*2*_ *O*	*Ethanol*
***Psiadia punctulata***						

*Alkaloids*	Wagner's test	-	-	**+**	**+**	**+**

*Tannins*	Ferric chloride test	-	-	**+**	**+**	**+**

*Saponins*	Foam test	-	-	-	**+**	**+**

*Flavonoids*	Alkaline reagent test	-	**+**	**+**	**+**	**+**

*Phytosterols*	Modified Liebermann-Burchard's test	**+**	-	-	-	-

***Meriandra bengalensis***						

*Alkaloids*	Wagner's test	+	+	+	+	+

*Tannins *	Ferric chloride test	-	-	+	+	+

*Saponins*	Foam test	-	-	-	+	+

*Flavonoids*	Alkaline reagent test	-	+	+	+	+

*Phytosterols*	Modified Liebermann-Burchard's test	+	+	+	-	-

(+) sign denotes the presence of corresponding phytoconstituents and (-) denotes the absence of corresponding phytoconstituents.

## Data Availability

Data will be available upon reasonable request to the corresponding author.
